# Patient and family-initiated escalation of care: a qualitative systematic review protocol

**DOI:** 10.1186/s13643-019-1010-z

**Published:** 2019-04-09

**Authors:** Aidín McKinney, Donna Fitzsimons, Bronagh Blackwood, Jennifer McGaughey

**Affiliations:** 1 School of Nursing and Midwifery, Queen’s University Belfast, 97 Lisburn Road, Belfast, BT9 7BL Northern Ireland; 2 Centre for Experimental Medicine, School of Medicine, Dentistry and Biomedical Sciences, Queen’s University Belfast, Wellcome-Wolfson Institute for Health Sciences, 97 Lisburn Rd, Belfast, BT9 7BL Northern Ireland

**Keywords:** Deterioration, Family-initiated rapid response, Family-initiated escalation of care, Hospital and experiences

## Abstract

**Background:**

Despite the introduction of rapid response systems and early warning scores, clinical deterioration that is not recognised or responded to early enough prevails in acute care areas. One intervention that aims to address this issue and that is gaining increased attention is patient- and family-initiated escalation of care schemes. Existing systematic review evidence to date has tended to focus on identifying the impact or effectiveness of these schemes in practice. However, they have not tended to focus on qualitative evidence to consider the experience of deterioration and the factors that may promote or hinder engagement with these schemes in the practice setting. This systematic review will address this gap. The aim of this review is to explore patients’, relatives’ and healthcare professionals’ experiences of deterioration and their perceptions of the barriers or facilitators to patient and family-initiated escalation of care in acute adult hospital wards.

**Methods:**

We will search Medline, CINAHL, Embase and PsycINFO databases using free-text and MESH terms relating to deterioration, family-initiated rapid response, families, patients, healthcare staff, hospital and experiences. We will search grey literature and reference lists of included studies for further published and unpublished literature. All studies with a qualitative design or method will be included. Two reviewers will independently assess studies for eligibility, extract data and appraise the quality of included studies. Data will be synthesised using a thematic synthesis approach, and findings will be presented narratively.

**Discussion:**

Patient- and family-initiated escalation of care schemes have been developed and implemented in several countries including the United States, the United Kingdom and Australia, but there is limited evidence regarding patients’ or families’ perceptions of deterioration or the barriers and facilitators to using these schemes in practice, particularly in acute adult areas. This systematic review will provide evidence for the development of a patient and family escalation of care scheme that can be tested in a feasibility study.

**Systematic review registration:**

PROSPERO CRD42018106952

**Electronic supplementary material:**

The online version of this article (10.1186/s13643-019-1010-z) contains supplementary material, which is available to authorized users.

## Background

### Description of the condition

Clinical deterioration can occur at any point in a patients’ illness. In particular, deterioration is more likely to occur following emergency admission, after surgery or after recovery from critical illness [1, 2]. The deteriorating patient has been defined as “one who moves from one clinical state to a worse clinical state which increases their individual risk of morbidity, including organ dysfunction, protracted hospital stay, disability, or death” p. 1033 [3]. Failure to recognise and respond to deterioration is an area of significant concern in today’s healthcare settings [1, 4, 5]. Clinical deterioration is commonly marked by patients exhibiting changes in vital signs prior to adverse events and may include physiological alterations in respiratory rate, conscious level, heart rate (HR) and/or blood pressure which are observed and recorded at the bedside [6, 7]. Prompt identification, interpretation and response to these physiological changes have been shown to improve patient outcomes, reduce intensive care unit (ICU) admissions and prevent adverse events [8–12]. A number of seminal studies however identified that care of the acutely ill patient can be suboptimal and that staff can miss or fail to appropriately manage patient deterioration [13–16].

In response to this, a number of systems have been implemented internationally in acute hospital settings to improve patient safety based on evidence-based guidelines [5, 17, 18]. The generic term used to describe these systems is “rapid response system” (RRS). These consist of a “crisis detection” mechanism that will trigger deterioration and a “response triggering” mechanism that will ensure that an individual or team will promptly respond to the trigger [19, 20]. However, despite the widespread implementation of RRS, patients have continued to deteriorate without being recognised or treated early enough. It has been identified that there continues to be a failure to correctly calculate scores, adequately respond to increasing early warning scores (EWS) [21] and adhere with escalation protocols [22–26]. Such failures to detect and refer patients have been attributed to organisational factors (workload, staffing levels and skill-mix), nurse experience associated with use of intuition and cultural factors (hierarchical communication) [23, 27–31]. Reliance on objective criteria to identify clinical deterioration may also be a limitation since they do not take into account the more subjective warning signs; however, Jones et al. [3] maintain that a significant number of rapid response team (RRT) calls may be the result of “concern” or a “worried” criterion. Odell et al. [32] emphasise that intuition needs to be considered as a valid way to detect clinical deterioration, reporting that nurses commonly identify deterioration through inductive reasoning. Similarly, for patients and relatives, while they may not necessarily be able to pick up on the more objective assessment methods employed to detect deterioration, it is possible that intuitively, they may also identify deterioration since they have greatest knowledge of the patient [33]. The contribution that patients and/or families can make to detect and manage acute deterioration is being highlighted more steadily [4], and families are now increasingly viewed as key stakeholders in detecting and responding to patient deterioration [5, 17, 18]. This has led to a number of patient and family-initiated escalation of care schemes being implemented internationally [5, 33, 34].

### Description of the intervention

A patient and family-initiated escalation of care scheme is an intervention whereby a patient or family member is facilitated within a hospital setting to raise concerns with healthcare professionals [34, 35]. Its purpose is to provide an avenue which will allow concerned patients and/or relatives to summon healthcare professionals to assess and respond to clinical deterioration that they remain worried about [36]. It serves as another safety mechanism so that patients who become acutely unwell may be identified early and managed on the ward in a timely manner, or else may be admitted to ICU at the right time rather than deteriorating further and developing potentially more complex care needs [36].

A number of patient and family-initiated escalation of care schemes currently exist in the United States (US), in the United Kingdom (UK) and in Australia [5, 33, 34, 37]. Condition HELP [34], REACH [38], Ryan’s Model [39] and Call4Concern [33] are amongst those identified to date. The aim of these various schemes is consistent in that they intend to facilitate concerned patients and/or relatives to seek help for clinical deterioration that they are concerned about; however, they seem to subscribe to different patient and relative-led escalation protocols [36]. Of the schemes that have been implemented, they appear to use two approaches to escalate care. Some implement an escalation of care scheme indirectly, such as Condition HELP, whereby the call for help is firstly triaged by the Condition HELP team who then determine whether help from a specialist team with high level of competency and skills such as the rapid response team (RRT) is required [34]. Other schemes appear to implement a more direct pathway with no triage step and allow the patient/relative to directly call the RRT themselves [33, 40]. Some of these also appear to incorporate a phased approach to call for help, e.g. REACH model and Call4Concern [33, 38], and advocate escalating care to the patients’ own care team initially but also include the option of facilitating patients/relatives to contact the RRT directly if they feel their concerns are not being adequately addressed [33, 38].

Further differences exist with regard to the composition of the RRT once activated and range from a nurse, nurse manager, doctor, critical care fellow and respiratory therapist [40] to a team consisting of a critical care nurse and respiratory therapist [41]. Confusion also arises from the variety of names that exist for the intervention. Family-initiated escalation of care [37], patient and family-initiated rapid response system [42], patient and relative-activated RRT [41] and family-initiated rapid response [43] are amongst those identified, with some appearing to promote both patient and family escalation [42] and others family escalation only [43]. It is therefore evident that greater clarity is needed in relation to what such systems/interventions should be called and who exactly they are intended for.

All schemes however appear to consist of three interrelated elements: a resource (informing patients/relatives of the scheme and how to use it), an identified method or source of help, and an identified response to the trigger [33, 34, 40]. The resources used to inform patients and relatives of the scheme and how to call for help may include educational booklets, information sessions, flyers, posters, DVDs or a combination of these [33, 34, 38]. The identified source of help that the patient may be directed to may include members of their own care team who they should speak to [38], a phone number to an operator or another individual who will triage the call [34] or a direct call or pager to members of the RRT [33]. An identified response to the trigger may include a review by the primary care team within 30 min of the call [38], a “call for help” team (but not the RRT) coming to review the patient [34] or may involve the RRT reviewing the patients’ condition [33].

### How the intervention might work

Patient and relative-initiated escalation of care schemes are based on the premise that the patient/relative intuitively knows their own or their relatives’ body best and therefore is best equipped to recognise any deviations in health status [44]. One theoretical framework that may help to provide a greater understanding of this viewpoint is the Common-Sense Model (CSM) of Self-Regulation by Leventhal and colleagues [45]. This starts from the premise that individuals tend to be active problem solvers who make sense to a threat to their health. It begins with analysing the processes by which patients become aware of a health threat, such as symptoms, developing their own cognitive representations to an illness and formulating perceptions to that threat, which in turn determines how they respond [45]. A novel feature of Leventhal’s model is to generate a greater understanding as to how people tend to process how they feel about the threat and what they might do in relation to it [46]. More specifically, the model identifies five key components that individuals use to understand their illness threat. These include developing an understanding of what the illness is and identifying its causes, its perceived timeline, its consequences and whether it can be cured or controlled [46]. This understanding may not necessarily be based on scientific or medical knowledge but may be formulated from previous personal experiences and interactions with others which may/or may not include healthcare providers [47]. These factors will all influence how patients may then seek self-care in relation to these.

This intervention also builds on current thinking that interventions work best and contribute to improvements in safety and quality, if there is active involvement with patients and families in their care [5, 38]. This includes involving patient and family in the early recognition and referral of deterioration which is increasingly recognised as a key area of research to improve patient safety in hospital [38].

A number of evaluations have been carried out to understand how these interventions work in practice. These have focused on identifying the number of patient and family-initiated calls, the reasons for activation and identifying who escalates calls [33, 34, 40, 41, 43, 48, 49]. In the majority of cases, most of the reasons for activating escalation of care were cited as appropriate [36, 50]. Bavare et al. [43] however would argue that there is a paucity of data that currently depicts reasons for which families activate RRT compared with those activated by clinicians. Eden et al. [42] also reported that calls tend to relate to non-safety issues and therefore questions the utility of patient and family-activated RRTs and whether they meet their intended purpose.

Evaluations have also sought to understand how effective these schemes are and their impact on patients, relatives, healthcare staff and the overall hospital environment. Evaluation of the schemes noted that relatives escalated a sub-set of patients missed by healthcare staff, identified a vital sign change that should have promoted clinical activation of RRS and a number of patients were transferred to higher dependency areas or received medical intervention on the ward [33, 40, 43, 50]. Findings also indicate that patients and/or families felt reassured when the service was available and reported high satisfaction levels with the schemes and increased feelings of empowerment [33, 34, 41]. Staff evaluations focused on identifying some of the concerns associated with patient and family involvement such as the possibility of inappropriate call activation and that it might lead to increased workload by staff [51]. However, the overall impact on hospital ward staff seemed to be minimal [33, 40]. This could be interpreted as positive as this intervention did not appear to impact significantly on staff time or resources; however, Albutt et al. [36] argue that it could also reflect unwillingness by both patients and relatives to participate in this scheme.

To synthesise the evidence in relation to the effectiveness and impact of the schemes, two recent systematic reviews by Albutt et al. [36] and Gill et al. [37] were identified. These highlight the overall poor quality and lack of evidence to determine the impact of patient and family-initiated schemes and therefore conclude that the recommendations for practice are limited [36, 37]. Albutt et al. [36] report that few studies were designed to establish the clinical effectiveness and that in some cases they were poorly designed. The majority of the included studies were descriptive quality improvement initiatives, audit or practice development evaluations which focused on evaluating the effectiveness of implementation strategies using non-clinical outcomes and identify that due to limited data it is difficult to determine impact [36, 37]. As a result, there was limited detail regarding the context of the intervention, ability of patients or families to recognise deterioration, how best to inform families of the process, agreement on outcome measures and limited reports of identifying family preferences to involvement [50]. There is therefore a need to further investigate and explore the assumptions that this scheme is based on, such as, can patients and relatives escalate care, is it a role they wish to take on and, if so, what factors may impact on any potential involvement [44].

### Why is it important to do this review?

This review is important as it will identify whether patients and/or relatives can detect deterioration and determine those barriers/facilitators to the implementation of patient and family-initiated schemes. Previous systematic reviews [36, 37] have tended to focus on the effectiveness and impact of the schemes on patients, relatives and healthcare professionals without considering the extent to which patients and relatives can detect deterioration. However, it is essential to address this further as it has important consequences for the implementation of such schemes as they rely on the assumption that patients/relatives can recognise clinical deterioration [36]. A synthesis of the qualitative evidence that explores this issue and explores the key barriers and facilitators to patient and family-initiated escalation of care from patients’, relatives’ and healthcare professionals’ perspectives may yield valuable insights that could inform the development of future patient and family escalation schemes. Interventions such as these are complex, and quantitative studies will not provide sufficient detail on the various factors that impact on their implementation. An understanding of such factors is however of significant importance and may contribute towards determining the success of such interventions in the future. However, to our knowledge, there is no qualitative systematic review that currently explores these factors particularly from acute adult settings. Such a review could help to explain the perspectives from various angles and add to our knowledge base and generate greater insights into why these interventions may succeed or fail. This information may then be used to influence the design and implementation of future patient and relative-initiated schemes to enhance their effectiveness.

## Methods

### Aim

This review aims to determine patients’, relatives’, and healthcare professionals’ experiences of deterioration and their perceptions of the barriers or facilitators to patient and family-initiated escalation of care in acute adult hospital wards.

### Objectives

1. To determine patients’ and relatives’ perceptions of their ability to recognise deterioration.
2. To identify patients’ perceptions of actual or potential barriers or facilitators to patient and family-initiated escalation of care schemes.
3. To explore and summarise relatives’ perceptions of actual or potential barriers or facilitators to patient and relative-initiated escalation of care schemes.
4. To summarise and outline healthcare professionals’ perceptions of actual or potential barriers or facilitators to patient and relative-initiated escalation of care schemes.

## Methods

This protocol was written in accordance with the Preferred Reporting Items for Systematic Reviews and Meta-Analyses Protocols (PRISMA-P) checklist (see Additional file 1).

### Types of studies

We will include any type of research design providing it has a qualitative element or includes a qualitative aspect within the design, such as mixed-methods research. This will include studies that employ qualitative study designs such as grounded theory, phenomenology, ethnography, action research and qualitative descriptive research. Any study that uses qualitative methods for data collection such as observation, individual and focus group interviews and qualitative methods for data analysis such as thematic analysis will also be included. We will exclude studies that analyse the data quantitatively. Published and unpublished data will be included.

### Types of participants

Acute adult patients, relatives and healthcare professionals (nurses, doctors, managers, educationalists and outreach staff) in acute hospital settings will be eligible for inclusion in this review. We will exclude studies reporting on the experiences or perspectives of patients, relatives, and healthcare staff from paediatric, maternity, mental health, non-acute wards, specialist areas (accident and emergency, intensive care unit, high-dependency unit) and community (non-hospital) settings.

### Phenomenon of interest

The primary phenomena of interest are the experiences and perceptions of patients, relatives and healthcare professionals relating to deterioration and/or towards patient and family-initiated escalation of care schemes. We will exclude healthcare professionals’ experiences or views of deterioration. We will also exclude patients’, relatives’ and healthcare professionals’ views of RRS not initiated by patients or relatives.

### Types of outcome

1. Perceptions of the indicators and extent to which patients/relatives can recognise deterioration in hospital.
2. Patient and family-reported perceptions of the barriers/facilitators to escalation of care in acute hospital ward settings.
3. Healthcare staffs’ perceptions of the factors that may facilitate or hinder the implementation of patient and family-initiated escalation of care schemes in the acute hospital ward setting.

### Search strategy

We will search Medline, CINAHL, Embase and PsycINFO databases using free-text and MESH terms related to deterioration, family-initiated rapid response, families, patients, healthcare staff, hospital and experiences with no language restrictions. The year of publication will be restricted from 2005 when the patient/family escalation of care schemes was implemented in practice. The search strategy will be developed with a subject librarian and piloted on Medline database (see Additional file 2). Subsequent searches will be adapted from the Medline strategy. A search of grey literature will include searching OpenGrey database, citation searching and contacting authors for further information or unpublished studies.

### Selection of studies

One author (AMcK) will screen titles and abstracts retrieved and exclude any studies that are irrelevant. Full text of potentially relevant articles will be retrieved, and two authors (AMcK, JMcG) will independently assess for inclusion based on study eligibility criteria. Any disagreements will be resolved by discussion and consensus reached or by independent third party arbitration (DF). No studies will be excluded on the basis of quality.

### Data extraction

Data extraction will be independently undertaken by two authors (AMcK, JMcG). A modified version of the EPOC data collection tool [52] which includes a full description of the intervention as per TIDieR checklist [53] will be used to extract data on the phenomenon of interest, characteristics of the intervention, population, setting, study design, methods and findings of significance to the review question and specific objectives (see Additional file 3). We will pilot the data extraction form and make changes if necessary prior to data extraction.

### Quality assessment

Two independent reviewers (AMcK, JMcG) will assess the methodological quality of included qualitative studies. We will use an adaptation of the Critical Appraisal Skills Programme [54] tool and Popay [55] framework for critical appraisal of qualitative studies as previously used by Jordan et al. [56] (see Additional file 3). The framework consists of ten questions designed to capture the quality of reporting methodological rigour and overall conceptual integrity of included studies. Each question will be scored as either yes, no or not reported and overall weighted as high (criteria was clearly applied and described in the paper or ascertained in communication with the primary author), moderate (criteria not reported clearly and unable to clarify with primary author) or low (criteria not applied or applied inappropriately) quality studies [56].

### Data synthesis

We will use Thomas and Harden’s thematic synthesis approach [57] which is one of the approaches considered appropriate for use in qualitative synthesis [58] and the Cochrane Qualitative and Implementation Methods Group (CQIMG) guidance on data extraction, synthesis and assessment of confidence [58]. Data synthesis will be undertaken with each relevant population group(s) for each outcome. This will be conducted collaboratively by two authors (AMcK, JMcG) in three rigorous stages. The first stage will consist of the reviewers independently reading each relevant article in detail and conducting line by line coding of the study findings from each of the primary studies using an inductive approach. This initial line by line coding will therefore involve capturing any reported participants’ interpretations of experiences (first-order concepts) as well as the authors’ interpretations (second-order concepts) [59]. The codes will then be constantly compared between studies, and differences and similarities between papers will be considered [60]. The next stage will involve organising or grouping these codes into related areas and constructing “descriptive” themes. The final stage of synthesis involves iteratively examining and comparing these descriptive themes across studies to refine the relationship between them and generate analytical themes in order to provide new insights related to the review question [57]. Study findings will be synthesised and presented narratively.

### Appraisal of certainty of review findings

To determine how much certainty can be placed in the qualitative evidence of each review finding, an assessment of the Confidence in the Evidence from Reviews of Qualitative research (CERQual) will be undertaken [61]. Four components contribute to the assessment of confidence, namely, methodological limitations, relevance, coherence and adequacy of data.

To provide an overall indication of the certainty of the qualitative evidence, studies will be assessed using four levels of confidence: high, moderate, low and very low. High confidence will indicate that the review finding is a reasonable presentation of the phenomenon of interest, moderate confidence indicates it is likely that the review is a reasonable representation of the phenomenon of interest, low confidence indicates it is possible that the review finding is a reasonable representation of the phenomenon of interest and very low confidence indicates that it is not clear whether the review finding is a reasonable representation of the phenomenon of interest. The application of CERQual to each review finding will be made independently by two review authors (AMcK, JMcG). Any concerns noted in relation to any of these identified components will lower confidence in the review finding. The assessment of confidence in the evidence will be summarised by themes and will be presented in the form of summary statements derived from synthesis of qualitative evidence (Additional file 4). This table is similar to the “Summary of findings” table used in Cochrane reviews of effectiveness and will summarise the key findings, our confidence in the evidence for each finding and an explanation in relation to how we arrived at our confidence of the evidence [62].

## Discussion

This qualitative review will systematically appraise and synthesise the research evidence related to patients’, relatives’ and healthcare professionals’ experiences of deterioration and their perceptions of the barriers and facilitators towards implementing patient and family-initiated escalation of care schemes in acute adult hospital settings. To our knowledge, this synthesis has not been carried out to date. Previous systematic reviews have focused on the effectiveness or impact of these schemes.

This systematic review is of relevance as it will provide an understanding of (1) the patients’/families’ perception of deterioration, (2) those factors that enable or constrain them in getting help while in hospital and (3) healthcare staffs’ perceptions of schemes to allow patients/families to escalate care. To date, there is limited understanding regarding patients’ or families’ perceptions of deterioration or the barriers and facilitators to using these schemes in practice. This systematic review will provide evidence for the development of a patient and family escalation of care scheme that can be tested in a feasibility study.

### Registration

This systematic review has been registered with PROSPERO (CRD42018106952), an international prospective register of systematic reviews [63].

## Additional files

Additional file 1: PRISMA-P 2015 checklist. (DOCX 36 kb)

Additional file 2: Medline search strategy. (DOCX 21 kb)

Additional file 3: Study eligibility, quality assessment and data extraction form. (DOCX 64 kb)

Additional file 4: Summary of qualitative findings table [59]. (DOCX 13 kb)

## Abbreviations

A&E: Accident and Emergency
CASP: Critical Appraisal Skills Programme
CERQual: Confidence in the Evidence from Reviews of Qualitative research
CSM: Common-Sense Model
EWS: Early Warning Scores
FIRRs: Family-initiated rapid response schemes
HDU: High-dependency unit
HR: Heart rate
ICU: Intensive care unit
PRISMA: Preferred Reporting Items for Systematic Reviews and Meta-Analyses
RRS: Rapid response system
RRT: Rapid response team
SoF: Summary of findings
UK: United Kingdom
US: United States
