# Supplementary material for: Patient and family-initiated escalation of care: a qualitative systematic review protocol
Source: Syst Rev. 2019 Apr 9;8:91. doi: 10.1186/s13643-019-1010-z (PMC6454605; doi:10.1186/s13643-019-1010-z)
Supplement: Supplementary file 2 — Medline search strategy. (DOCX 21 kb) [file 13643_2019_1010_MOESM2_ESM.docx]

**Additional File 2: Medline search strategy**

Database(s): **Ovid MEDLINE(R) ALL**1946 to July 18, 2018 

| **#** | **Searches** |
| --- | --- |
| 1 | "deterioration".mp. or CLINICAL DETERIORATION/ |
| 2 | "clinical deterioration".mp. or Clinical Deterioration/ |
| 3 | "deteriorating patient*".mp. |
| 4 | Hospital Rapid Response Team/ or "family-initiated rapid response".mp. |
| 5 | Hospital Rapid Response Team/ or "family-initiated rapid response system*".mp. |
| 6 | "family-initiated escalation of care".mp. or Hospital Rapid Response Team/ |
| 7 | "family-activated escalation of care".mp. [mp=title, abstract, original title, name of substance word, subject heading word, floating sub-heading word, keyword heading word, protocol supplementary concept word, rare disease supplementary concept word, unique identifier, synonyms] |
| 8 | "calling for help".mp. |
| 9 | "condition help".mp. |
| 10 | "medical emergency team*".mp. |
| 11 | "rapid response system".mp. |
| 12 | 1 or 2 or 3 or 4 or 5 or 6 or 7 or 8 or 9 or 10 or 11 |
| 13 | Family/ or "famil*".mp. |
| 14 | "relativ*".mp. |
| 15 | "patients".mp. or PATIENTS/ |
| 16 | Health Personnel/ or Nursing Staff, Hospital/ or "healthcare staff".mp. |
| 17 | "nurse*".mp. |
| 18 | "doctor*".mp. or Physicians/ |
| 19 | "medical staff".mp. or Medical Staff, Hospital/ or Medical Staff/ |
| 20 | "critical care outreach team".mp. |
| 21 | 13 or 14 or 15 or 16 or 17 or 18 or 19 or 20 |
| 22 | "hospital".mp. or Hospitals/ |
| 23 | "general ward*".mp. |
| 24 | 22 or 23 |
| 25 | "experience".mp. |
| 26 | "views".mp. |
| 27 | "opinions".mp. or Attitude/ |
| 28 | "perceptions".mp. or Perception/ |
| 29 | ATTITUDE/ or "attitude".mp. |
| 30 | 25 or 26 or 27 or 28 or 29 |
| 31 | 12 and 21 and 24 and 30 |
| 32 | limit 31 to yr="2005 -Current" |
