# Supplementary material for: Patient and family-initiated escalation of care: a qualitative systematic review protocol
Source: Syst Rev. 2019 Apr 9;8:91. doi: 10.1186/s13643-019-1010-z (PMC6454605; doi:10.1186/s13643-019-1010-z)
Supplement: Supplementary file 3 — Study eligibility, quality assessment and data extraction form. (DOCX 64 kb) [file 13643_2019_1010_MOESM3_ESM.docx]

**Additional File 3**

**Study Eligibility, Quality Assessment & Data Extraction Form**

Name of author extracting data: _____________________________________

Date form completed: _____________________________________

**Study ID**

| Title |  |
| --- | --- |
| Study ID  (Family name of first author and year of publication and letter if more than one year, e.g. McKinney 2018a) |  |
| Are there other articles of same?  (Yes, No, Unclear. If Yes, write study IDs) |  |

**Study Eligibility**

| **Inclusion criteria met?** | | | |
| --- | --- | --- | --- |
| **Type of studies**  1. Is this a qualitative study?  2. Does this study have a qualitative element? | **Yes, Unclear, No**  **Yes, Unclear, No** | **Source (pg no.):** | |
|  | **Notes:** | | |
| **Types of participants/Phenomena of Interest**  1. Adult patients who had experience of acute deterioration and/or patient/family-initiated escalation of care?  2. Relatives of adult patients who had experience of acute deterioration and/or patient/family-initiated escalation of care?  3. Healthcare professionals who had experience/views of adult patient/family-initiated escalation of care? | **Yes, Unclear, No**  **Yes, Unclear, No**  **Yes, Unclear, No** | **Source (pg no.):** | |
|  | **Notes:** | | |
| **Setting:**  Acute hospital ward | **Yes, Unclear, No** | | **Source (pg no.):** |
|  | **Notes:** | | |
| **Conclusion**   - **Included** - **Excluded and listed in excluded table** - **More information needed before inclusion decision (specify)** | | | |

**Data Extraction Form**

**Design**

| Type of Design |  |
| --- | --- |
| Was the study conducted as… | - Stand-alone qualitative study - Part of a larger qualitative study - Part of mixed methods study |
| Focus of study | - Experiences of deterioration - Patient and family-initiated escalation of care schemes - Mixed (please specify) - Other (please specify) |
| Aims & Objectives |  |
| Phenomena of interest e.g. types of deterioration/escalation of care ie characteristics |  |
| Setting | - University Teaching Hospital - Private Hospital - Public Hospital - Non-teaching hospital - Urban setting - Rural setting - Tertiary - Other (Please state)_________________________     Type of Ward:_________________________________ |
| Country and/or participating sites |  |

**Study population**

| Total Participants Recruited |  |
| --- | --- |
| Healthcare Professionals  Years experience ______  Grade ______    Number of nurses ______  Number of doctors ______  Number of other ______ | Patients and relatives |
|  | - Patient - Family member   Numbers  Patients _________  Relatives _________ |
| Method of selection e.g purposive/convenience sampling |  |
| Inclusion/Exclusion criteria |  |

**Theoretical/ conceptual framework**

| Stated framework/orientation | - Yes - No   If Yes, Please state type:___________________ |
| --- | --- |
| Detail provided re: chosen framework |  |

**Data collection**

| Method of data collection | - Unstructured - Semi-structured individual interviews - Focus groups - Participant observation - Non-participant observation - Other (please specify) ____________ | | | |
| --- | --- | --- | --- | --- |
| Method of data recording | - Written - Digital recording - Video recording - Other (please specify) ____________ | | | |
| Study duration and follow up |  | | | |
| **Reporting of Intervention as per TIDier checklist [53]** | | | | |
| 1.Name of intervention: |  | | | |
| 2.Goal of intervention: |  | | | |
| 3.Materials used: |  | | | |
| 4.Procedures/activities used: |  | | | |
| 5.Details on intervention provider: (expertise, training given) |  | | | |
| 6.Describe modes of intervention delivery: |  | | | |
| 7.Location of intervention: |  | | | |
| 8.Number of time intervention was delivered, over what time period: |  | | | |
| 9.Was intervention planned to be personalised? |  | | | |
| 10.Was intervention modified? |  | | | |
| 11.Strategies used to ensure fidelity (intervention implemented as intended): |  | | | |
| 12.Was intervention delivered as planned? |  | | | |
| **Tick yes or no:** | | | | |
| Did hospitals have EWS? | Yes |  | No |  |
|  | If yes, provide details. For eg state type of EWS used below: | | | |
| Was there a facility to record patients/relatives concerns on EWS chart? | Yes |  | No |  |
|  | If yes, provide details: | | | |
| Did hospitals have CCOT/RRT/MET? | Yes |  | No |  |
|  | If yes, provide details. For eg on the composition/ availability of the team: | | | |
| Were patients/relatives able to trigger or self refer to CCOT/RRT/MET? | Yes |  | No |  |
|  | If yes, provide details: | | | |

**Data analysis**

| Method of data analysis (tick all that apply) | - Thematic content analysis - Grounded theory - Discourse analysis - Narrative analysis - Other (please specify) |
| --- | --- |
| Procedures for data analysis e.g. use of computer software package, process(es) of coding? | |
| Details provided re: chosen data analysis method | |

**Outcomes**

| Experiences of deterioration |  |
| --- | --- |
| Barriers to Patient/family-initiated Escalation of Care |  |
| Facilitators to Patient/family-initiated Escalation of Care |  |
| Was fidelity, dose and reach of intervention discussed? | |
| Other outcomes that are reported – that could provide additional insight | |

**Summary**

| Summary of key findings (by paraphrasing of use of quotes) |
| --- |
| Author(s) inferences/ implications for practice/policy |
| Author(s) conclusions |
| Any other issues/ comments |
| Limitations |

**Quality assessment (Jordan et al, 2016)**

| **Question / Criteria** | **Assessment** |
| --- | --- |
| Is there a logical fit between stated research aim(s) and method(s) used? | ’Yes’, ’No’ or ’Not reported’ |
| Is the recruitment strategy appropriate to the aims of the research? | ’Yes’, ’No’ or ’Not reported’ |
| Is there detailed evidence of steps taken in data collection (e.g. interview guide, means of recording, how focus group composed) and why? | ’Yes (full/minimal detail)’, ’No’ or ’Not reported’ |
| Were the data audio-recorded and transcribed? | ’Yes’, ’No’ or ’Not reported’ |
| Is there a detailed statement of steps taken in data analysis? | ’Yes (full/minimal detail)’, ’No’ or ’Not reported’ |
| Did data analysis involve inter-rater discussion? | ’Yes’, ’No’ or ’Not reported’ |
| Was there consideration of disconfirming findings? | ’Yes’, ’No’ or ’Not reported’ |
| Is there a clear and detailed statement of findings? | ’Yes’, ’No’ or ’Not reported’ |
| Is there evidence of a reflexive concern with the conduct of the study? | ’Yes’, ’No’ or ’Not reported’ |
| Is there evidence of analysis and interpretation of the findings at a conceptual and theoretical level? | ’Yes’, ’No’ or ’Not reported’ |
| Summary quality assessment | ’High’, ’Moderate’ or ’Low’ |
