# Supplementary material for: Patient and family-initiated escalation of care: a qualitative systematic review protocol
Source: Syst Rev. 2019 Apr 9;8:91. doi: 10.1186/s13643-019-1010-z (PMC6454605; doi:10.1186/s13643-019-1010-z)
Supplement: Supplementary file 4 — Summary of qualitative findings table [59]. (DOCX 13 kb) [file 13643_2019_1010_MOESM4_ESM.docx]

**Additional File 4: Summary of Qualitative Findings Table [59]**

| **Review Finding** | **CERQual Assessment of Confidence in the Evidence** | **Explanation of CERQual Assessment** | **Studies contributing to the Review Findings** |
| --- | --- | --- | --- |
|  |  |  |  |
